# Beta‐cell death and dysfunction drives hyperglycaemia in organ donors

**DOI:** 10.1111/dom.15248

**Published:** 2023-08-30

**Authors:** Iestyn M. Shapey, Angela Summers, James O'Sullivan, Catherine Fullwood, Neil A. Hanley, John Casey, Shareen Forbes, Miranda Rosenthal, Paul R. V. Johnson, Pratik Choudhary, James Bushnell, James A. M. Shaw, Daniel Neiman, Ruth Shemer, Benjamin Glaser, Yuval Dor, Titus Augustine, Martin K. Rutter, David van Dellen

**Affiliations:** ^1^ Faculty of Medicine, Biology and Health University of Manchester Manchester UK; ^2^ Department of Renal and Pancreatic Transplantation Manchester University NHS Foundation Trust, Manchester Academic Health Science Centre, NIHR Manchester Biomedical Research Centre Manchester UK; ^3^ Manchester Centre for Genomic Medicine Manchester University NHS Foundation Trust Manchester UK; ^4^ Department of Research and Innovation (medical statistics) Manchester University NHS Foundation Trust, Manchester Academic Health Science Centre Manchester UK; ^5^ Transplant Unit, Royal Infirmary of Edinburgh Edinburgh UK; ^6^ Endocrinology Unit, University of Edinburgh Edinburgh UK; ^7^ Diabetes Unit, Royal Free Hospital London UK; ^8^ Oxford Centre for Diabetes, Endocrinology and Metabolism University of Oxford Oxford UK; ^9^ Diabetes Research Group, King's College London London UK; ^10^ Richard Bright Renal Unit, Southmead Hospital Bristol UK; ^11^ Institute of Cellular Medicine Newcastle University Newcastle UK; ^12^ Department of Developmental Biology and Cancer Research, Institute for Medical Research Israel‐Canada The Hebrew University‐Hadassah Medical School Jerusalem Israel; ^13^ Department of Endocrinology and Metabolism, Hadassah Medical Center and Faculty of Medicine Hebrew University of Jerusalem Jerusalem Israel; ^14^ Diabetes, Endocrinology and Metabolism Centre Manchester University NHS Foundation Trust, Manchester Academic Health Science Centre Manchester UK

**Keywords:** beta‐cell death, glycaemic control, hyperglycaemia, insulin, islet, organ donor, pancreas, transplant

## Abstract

**Background:**

Donor hyperglycaemia following brain death has been attributed to reversible insulin resistance. However, our islet and pancreas transplant data suggest that other mechanisms may be predominant. We aimed to determine the relationships between donor insulin use and markers of beta‐cell death and beta‐cell function in pancreas donors after brain death.

**Methods:**

In pancreas donors after brain death, we compared clinical and biochemical data in ‘insulin‐treated’ and ‘not insulin‐treated donors’ (IT vs. not‐IT). We measured plasma glucose, C‐peptide and levels of circulating unmethylated insulin gene promoter cell‐free DNA (*INS‐*cfDNA) and microRNA‐375 (miR‐375), as measures of beta‐cell death. Relationships between markers of beta‐cell death and islet isolation outcomes and post‐transplant function were also evaluated.

**Results:**

Of 92 pancreas donors, 40 (43%) required insulin. Glycaemic control and beta‐cell function were significantly poorer in IT donors versus not‐IT donors [median (IQR) peak glucose: 8 (7‐11) vs. 6 (6‐8) mmol/L, *p* = .016; C‐peptide: 3280 (3159‐3386) vs. 3195 (2868‐3386) pmol/L, *p* = .046]. IT donors had significantly higher levels of *INS*‐cfDNA [35 (18‐52) vs. 30 (8‐51) copies/ml, *p* = .035] and miR‐375 [1.050 (0.19‐1.95) vs. 0.73 (0.32‐1.10) copies/nl, *p* = .05]. Circulating donor miR‐375 was highly predictive of recipient islet graft failure at 3 months [adjusted receiver operator curve (SE) = 0.813 (0.149)].

**Conclusions:**

In pancreas donors, hyperglycaemia requiring IT is strongly associated with beta‐cell death. This provides an explanation for the relationship of donor IT with post‐transplant beta‐cell dysfunction in transplant recipients.

## 1 INTRODUCTION

Hyperglycaemia develops in approximately 50% of deceased organ donors on critical care. It is managed with insulin^1^ and is commonly attributed to insulin resistance.^2^ However, there are limited data on the underlying pathophysiology of hyperglycaemia in this setting. A 1993 study by Masson et al. of 25 human organ donors after brain death (DBD; defined as apnoeic coma with absence of brain‐stem reflexes), showed that hyperglycaemia, with elevated C‐peptide levels, was seen in 16 of these patients and that glucose levels could not be normalized with insulin in one‐third of the cohort.^3^ Hyperglycaemia during organ donation has therefore been attributed to insulin resistance, caused by a surge in catecholamine and inflammatory cytokine release. However, it remains unclear how much other explanations such as irreversible beta‐cell death contribute to hyperglycaemia in organ donors on intensive care units.

Our contemporary clinical data from over 2000 pancreas transplants have shown that donors requiring insulin had dose‐dependent higher risks of isolated islet failure 3 months post‐transplant, when compared with pancreas recipients from donors not treated with insulin.^1^ Likewise, our data from 91 islet transplants recipients showed that beta‐cell function 3 months post‐transplant was lower when islets were transplanted from donors requiring insulin compared with donors not requiring insulin.^4^ These findings remained unchanged after adjusting for potential confounders including donor age, body mass index, glucocorticoid use and cold ischaemic time. Moreover, peri‐donation glycated haemoglobin levels showed that donor insulin use in pancreas donors was not explained by pre‐existing undiagnosed diabetes. Our clinical findings are not commensurate with the theory that reversible insulin resistance is the sole explanation for donor hyperglycaemia and, consequently, impaired islet function post‐transplantation.

Recent technological advances have enabled detection of tissue specific circulating biomarkers of beta‐cell death such as cell‐free microRNA‐375 (miR‐375) and unmethylated DNA from the *insulin* (*INS*) gene promoter.^5^, ^6^, ^7^ miR‐375 is differentially expressed in pancreatic islets and is required for their development.^8^ Meanwhile, the INS gene promoter is unmethylated (active) solely in pancreatic beta cells, whereas it is methylated when derived from other cell types.^6^ Plasma levels of unmethylated INS gene promoter are low in health because it is intracellular.

We hypothesized that hyperglycaemia related to donor insulin use in DBDs organ donors is largely caused by beta‐cell death. Our aim was to determine the relationships of donor insulin use with tissue‐specific biomarkers of beta‐cell death and beta‐cell function in DBDs organ donors.

## 2 METHODS

### 2.1 Patient cohorts and samples

Data on organ donors from the UK Transplant Registry, held by NHS Blood and Transplant (NHSBT), were linked to plasma samples from a cohort of organ DBD recruited via the Quality in Organ Donation (QUOD) Biobank. Donors with pre‐existing diabetes mellitus were excluded as they were ineligible to donate a pancreas for transplantation. Plasma samples were collected in EDTA tubes at the time of aortic‐cross clamping during organ retrieval and represent the final point of normothermic circulation. Samples were centrifuged at 1300RCF for 15 min and plasma was stored at −80°C. To assess the relationship between donor data, donor biomarkers and clinical outcomes following islet transplantation, data from QUOD, NHSBT and the UK Islet Transplant Consortium (UKITC) sources were linked at the individual patient level.

### 2.2 MicroRNA assays

MicroRNA was extracted from 0.5 ml of plasma using the miRNeasy serum/plasma kit (Qiagen) and underwent reverse transcription using the miScript II RT kit (Qiagen) and pre‐amplification using the miScript PreAMP polymerase chain reaction (PCR) kit (Qiagen). Beta‐cell death was assessed from expression of miR‐375 using digital droplet PCR for absolute quantification (QX200; Bio‐Rad) miR‐375 assays were further standardized by measuring levels relative to a stable house‐keeping gene (SNORD‐95) using miScript Primer Assays SYBR Green RT PCR kits (Qiagen). All assays were performed in duplicate with mean values presented. Other miR associated with beta‐cell death (miR‐34a, 200b, 200c and 21) were also measured using miScript Primer Assays SYBR Green RT PCR kits (Qiagen).

### 2.3 Unmethylated insulin gene promoter cell‐free DNA assays

cfDNA was extracted robotically from 0.5 ml of plasma using QIAsymphony (Qiagen) and bisulphite treated. CpG sites that are uniquely unmethylated in beta cells were selected as previously described.^6^, ^9^ cfDNA was amplified by PCR as described before and pooled PCR products were analysed using a NextSeq sequencer (Illumina). Absolute values of beta‐cell cfDNA were calculated by multiplying the fraction of beta cell‐derived molecules by the total concentration of cfDNA present in the sample, as measured by Qubit. Data from organ donors were normalized against data from 30 healthy individuals.

### 2.4 Glucose and immunoassays

Glucose levels were measured at multiple time points, as determined clinically, and analysed using calibrated point‐of‐care analysers in critical care units at local donor hospitals. Peak donor glucose was defined as the highest recorded value during the donation phase. Given the limitations of current donor pancreas assessment, peak donor glucose and the administration of variable rate insulin infusions were considered the most reliable and reproducible metrics of glycaemic control following brain death. Magnetic Luminex microparticle multiplex assays (R&D Systems) were used to measure C‐peptide (lower limits of detection: 10 pmol/L), Fas ligand (1.2 pg/ml), Granzyme B (1.42 pg/ml), interleukin (IL)‐1 (0.8 pg/ml), IL‐6 (1.7 pg/ml), tumour necrosis factor α (1.2 pg/ml) and tumour necrosis factor‐related apoptosis‐inducing ligand (9.7 pg/ml). All assays were performed in duplicate with mean values presented.

### 2.5 Islet, isolation, assessment and transplantation

Pancreases were transported at 4°C in University of Wisconsin solution to one of three human islet isolation facilities located in Edinburgh, London or Oxford. Islets were isolated using standard procedures for human islet isolation comprising enzymatic digestion and mechanical dissociation within a Ricordi‐type digestion chamber, followed by density gradient purification using a continuous gradient on a COBE 2991 cell processor.^10^, ^11^ Islet viability was determined by a fluorometric fluorescein diacetate and propidium iodide assay, which stained viable cells green and dead cells red. Islet equivalent numbers (IEQ) were derived from counting and measuring the number of total islets in dithizone‐stained aliquots of purified samples and converted into the equivalent number of an idealized islet diameter of 150 μm. Islets were cultured for at least 24 h and released for transplantation if the following criteria were met: islet mass >3000 IEQ/kg recipient body weight; viability >70%; and purity >50%. When the allocated recipient was located at a different unit to the isolation centre, islets were resuspended in 500 ml of CMRL medium (PAA Laboratories Ltd) supplemented with 2% human serum albumin (ZENLAB20; Bio Products Laboratories) and 2 mM HEPES (PAA Laboratories Ltd) and transported by road in cooled standard blood transfusion bags.^10^ Islets were transplanted by radiologically guided percutaneous trans‐hepatic infusion into the portal vein or in exceptional cases via cannulation of the mesenteric vein following mini‐laparotomy.

### 2.6 Standardizing insulin therapy in organ donors

The UK NHSBT donor care bundle provides standardized guidance to maintain donor glucose levels between 4 and 10 mmol/L and to start a variable rate insulin infusion at a minimum rate of 1 U/h when appropriate.^12^ Donor insulin therapy (IT) was defined as any requirement for exogenous insulin during the peri‐donation period. The duration of insulin use was also considered, but data on total insulin dosage were not available.

### 2.7 Defining graft function and failure

Following islet transplantation, graft function was determined by fasting and 90‐min stimulated C‐peptide and glucose levels following a mixed meal tolerance test assessed at 3 months post‐transplantation.^13^ Islet graft failure was defined as a stimulated C‐peptide of <50 pmol/L. Graft failure following pancreas transplantation was defined as a return to exogenous IT.

### 2.8 Statistical methods

We assessed the distribution of donor variables for exposures and covariates according to donor insulin use. Donor glucose levels and donor C‐peptide were measured according to donor insulin use. Analyses were performed using Fisher's exact test, Student's t‐test or the Mann‐Whitney U‐test as appropriate.

Circulating donor *INS*‐cfDNA and *miR*‐375 were related to markers of donor glycaemic control and beta‐cell function using univariable linear regression analysis. These data corroborated with previous findings showing the tissue‐specificity of the assays.^6^, ^8^ Levels of *INS*‐cfDNA (absolute values and percentage of total DNA), and miR‐375 were compared according to donor insulin use using the Mann‐Whitney U‐test.

Donor levels of apoptosis markers and inflammatory cytokines were compared according to insulin use using the Mann‐Whitney U‐test; and according to donor beta‐cell death and beta‐cell function using univariable linear regression.

Statistical analysis was performed using SPSS (IBM SPSS Statistics for Windows, Version 22.0, 2013 release). Statistical significance was assumed when *p* < .05.

Our preliminary data in a small (*n* = 46) cohort of pancreas transplant recipients showed mean (SD) C‐peptide levels immediately post‐reperfusion of 3.1 (3.0) ng/ml in donors requiring insulin and 5.8 (3.8) ng/ml in those not requiring insulin. We calculated that a sample size of 50 in each group would have 94% power (*p* < .01) to detect a clinically significant difference in beta‐cell function and C‐peptide. In a regression analysis including five additional covariates in addition to data on insulin, the study would have 80% power (*p* < .05) to detect a 15% difference in C‐peptide levels.

### 2.9 Ethics statement

All data were collected prospectively, with ethical approval (QUOD 13/NW/0017; UKITC 07/Q0904/11), and with written informed consent from all donor families and transplant recipients, and in line with the Helsinki declaration.

## 3 RESULTS

In 92 pancreas donors [49 (53%) female; median (IQR) age 35 (25‐49) years, body mass index 24 (22‐26) kg/m^2^], 40 (43%) required insulin on intensive care. There were no significant differences in donor clinical variables according to treatment with IT (Table 1).

**TABLE 1:** Donor characteristics associated with donor insulin use on intensive care in pancreas donors

Donor variables	Insulin (n = 40)	No insulin (n = 52)	*p*‐Value
Age, years^a^	38 (13)	35 (14)	.310
BMI,^a^ kg/m^2^	23 (2.6)	24 (3.6)	.075
Female sex	20 (50)	29 (56)	.675
Ethnicity			
White	35 (78)	45 (87)	.573
Asian	3 (8)	3 (6)	.529
Black	0 (0)	2 (4)	.316
Other	2 (5)	2 (4)	.586
Cardiac arrest	13 (33)	14 (27)	.646
Hypotension	27 (68)	34 (65)	>.999
Steroid use	33 (64)	36 (83)	.224
Smoking	23 (58)	24 (46)	.301
Alcohol excess	5 (13)	3 (8)	.222
Hypertension	4 (6)	3 (8)	.463
Cardiac disease	3 (8)	0 (0)	.079
Cause of death			
ICH	21 (53)	29 (56)	.685
HBI	8 (20)	10 (19)	>.999
Trauma	5 (13)	9 (17)	.572
Other	6 (15)	4 (8)	.321
Peak donor glucose, mmol/L	8 (7‐11)	6 (6‐8)	.016
miR‐375, copies/nl	1.05 (0.12‐1.95)	0.73 (0.32‐1.00)	.05
*INS*‐cfDNA, copies/ml	35 (18‐52)	30 (8‐51)	.035

*Note*: Data are n (%) or median (IQR) unless stated.

Abbreviations: BMI, body mass index; ICH, intracranial haemorrhage; INS‐cfDNA, circulating cell‐free unmethylated DNA from the INS gene promotor; HBI, hypoxic brain injury; miR‐375, circulating cell‐free microRNA‐375.

^a^ Mean (SD).

Donor glycaemic control was worse in IT donors compared with not‐IT donors [median (IQR) peak glucose IT donors vs. not‐IT donors: 8 (7‐11) vs. 6 (6‐8) mmol/L, *p* = .016 Table 1]. IT donors had higher levels of *INS*‐cfDNA [35 (18‐52) vs. 30 (8‐51) copies/ml, *p* = .035] and miR‐375 [1.1 (0.19‐1.95) vs. 0.7 (0.32‐1.10) copies/nl, *p* = .050]. There was no difference in the total concentration of cfDNA between insulin and no‐insulin groups [median (IQR): 80 (52‐105) vs. 110 (54‐168) ng/ml, *p* = .088]. However, donor *INS‐*cfDNA, as a fraction of total cell‐free DNA, was higher in IT vs. not‐IT groups [median (IQR)% 0.14 (0.09‐0.27) vs. 0.09 (0.04‐0.15), *p* = .013].

Higher levels of circulating cell‐free *miR*‐375 levels were related to higher donor blood glucose levels [β (SE) 0.30 (0.16), *p* = .047], higher donor C‐peptide levels [688 (281), *p* = .017; Table 2]. Higher levels of donor *INS*‐cfDNA were related to higher donor C‐peptide levels [β (SE): 683 (287), *p* = .020].

**TABLE 2:** Relationships of donor markers of beta‐cell death with measures of glycaemic control, beta‐cell function and islet isolation outcomes in the donor, and beta‐cell function in islet transplant recipients

Variable	N	miR‐375		*INS*‐cfDNA	
		β (SE)	*p*‐Value	β (SE)	*p*‐Value
Peak donor blood glucose	92	0.30 (0.16)	.047	0.04 (0.08)	.599
Donor C‐peptide	92	688 (281)	.017	683 (287)	.020
Islet viability	16	−0.31 (0.11)	.014	0.01 (0.01)	.206
Islet yield	16	16.59 (24.63)	.513	−0.38 (0.18)	.05
Recipient stimulated C‐peptide^a^	12	−202 (87)	.043	0.16 (0.29)	.597

*Note*: Data are β (SE) from linear regression.

Abbreviations: INS‐cfDNA, circulating cell‐free unmethylated DNA from the INS gene promotor; miR‐375, circulating cell‐free microRNA‐375.

^a^ Relates to mixed meal tolerance tests at 3 months post islet transplantation in transplant naïve recipients.

Sixteen donors proceeded to successful islet isolation and islet transplantation (12 naïve) (Table 3). Recipients of islet transplants were predominately female: *n* = 14 (88%); aged 49 (39‐59), with body mass index 24 (21‐28) kg/m^2^. In the 16 donors proceeding to successful islet isolation, the median (IQR) islet yield was 375 (312‐457) × 1000 IEQ, viability was 87 (81‐92)% and purity 78 (71‐85)%; donor and recipient variables were similar according to donor insulin use. Three (25%) of the 12 islet transplants that were the recipients' first islet transplant failed within 3 months, two of which were from donors requiring insulin. The remaining 76 donors proceeded to pancreatic transplantation, of which six failed within 3 months post‐transplantation: thrombosis (*n* = 1), anastomotic leak (*n* = 1), pancreatitis (*n* = 1), isolated islet failure (*n* = 3). Further analysis of the pancreatic transplantation subgroup was inhibited by heterogeneity of the causes of failure.

**TABLE 3:** Relationships of donor and recipient variables and markers of beta‐cell death according to insulin use in the donor in islet transplant recipients

Variables	Insulin (n = 5)	No insulin (n = 11)
Donor		
Age, years^a^	46 (10)	45 (10)
BMI,^a^ kg/m^2^	25 (1.8)	29 (2.8)
Female sex	5 (100)	9 (82)
Ethnicity		
White	5 (100)	11 (100)
Cardiac arrest	1 (20)	2 (18)
Hypotension	4 (80)	7 (64)
Steroid use	4 (80)	9 (82)
Smoking	1 (20)	1 (9)
Alcohol excess	0	3 (27)
Hypertension	0	1 (9)
Cardiac disease	0	1 (9)
Cause of death		
ICH	3 (60)	8 (73)
HBI	0 (0)	2 (18)
Trauma	1 (20)	0 (0)
Other	1 (20)	1 (9)
miR‐375, copies/nl	1.2 (0.55‐2.70)	1.14 (0.16‐6.05)
*INS*‐cfDNA, copies/ml	49.0 (31.1‐214.7)	6.02 (27.9‐249.34)
Recipient		
Age, years^a^	55 (13)	47 (10)
BMI,^a^ kg/m^2^	24 (4.0)	25 (3.2)
Female sex	5 (100)	9 (82)
Ethnicity		
White	5 (100)	11 (100)
Islet Isolation outcomes		
Total yield, IEQ	388 (310‐443)	370 (307‐490)
Viability, %	85 (83‐93)	89 (80‐92)
Purity, %	85 (63‐90)	75 (70‐85)

*Note*: Data are n (%) or median (IQR) unless stated.

Abbreviations: BMI, body mass index; HBI, hypoxic brain injury; ICH, intracranial haemorrhage; IEQ, islet equivalents × 1000; INS‐cfDNA, circulating cell‐free unmethylated DNA from the INS gene promotor; miR‐375, circulating cell‐free microRNA‐375.

^a^ Mean (SD).

**FIGURE 1:**
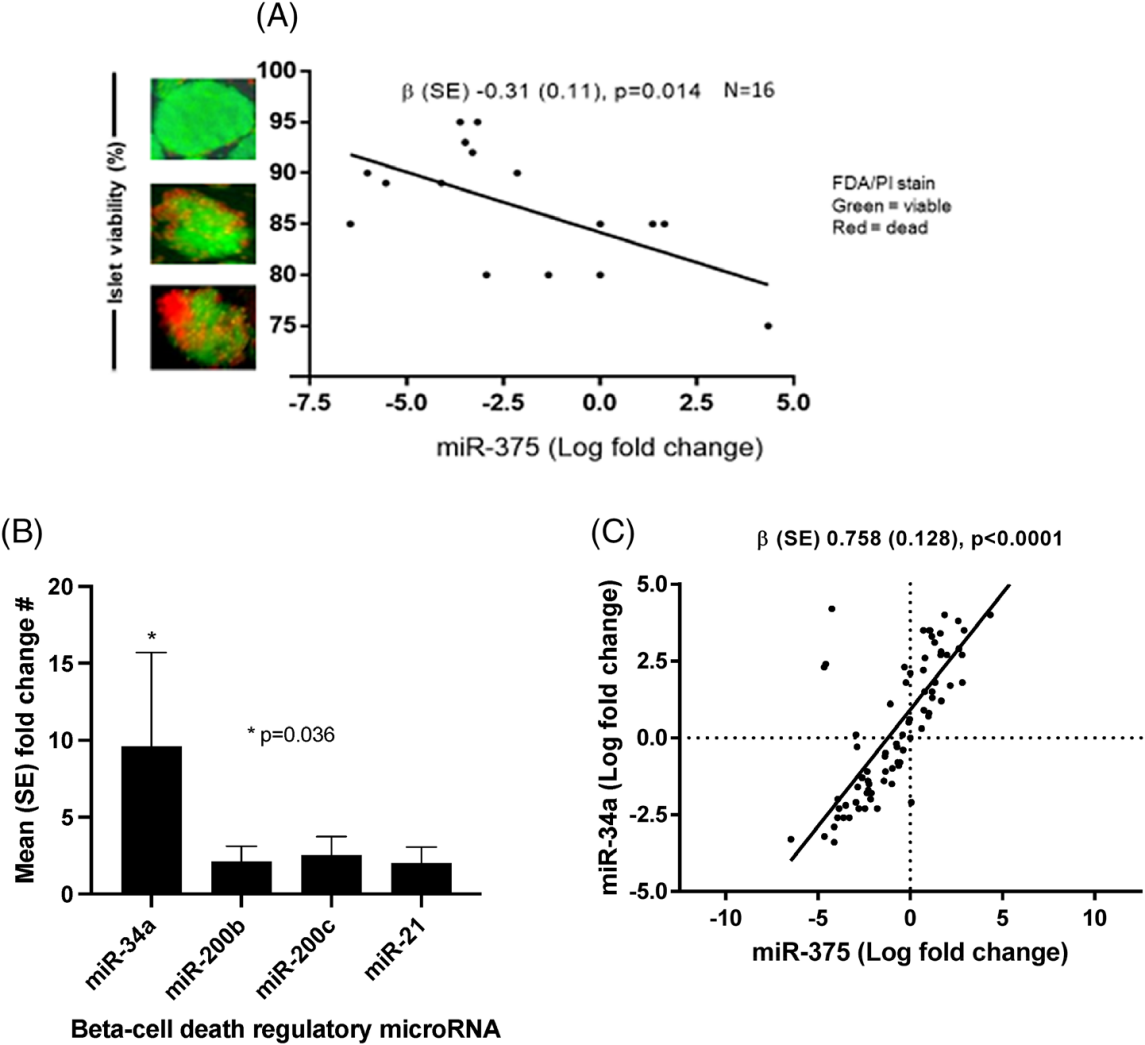
Beta‐cell death‐related circulating cell‐free microRNA in pancreas donors showing: A, relationship between levels of circulating cell‐free miR‐375 and islet viability, as assessed by linear regression; B, differences in circulating cell‐free miRNA levels comparing donors treated with and without insulin therapy on intensive care at the time of cross‐clamp during organ donation; C, relationship between levels of circulating cell‐free miR‐375 and miR‐34a, as assessed by linear regression.

In donors providing pancreases that went for islet isolation, higher levels of circulating cell‐free miR‐375 levels in donors were related to lower islet viability [−0.31 (0.11), *p* = .01], but not islet yield (Figure 1A; Table 3). In islet transplant recipients, donor miR‐375 was related to both lower recipients fasting C‐peptide levels [−72 (29), *p* = .043] and lower recipient stimulated C‐peptide levels [−202 (87), *p* = .046] 3 months following islet transplantation.

Higher levels of donor *INS*‐cfDNA did not predict stimulated C‐peptide in islet transplant recipients. There was a borderline significant relationship between higher levels of donor *INS*‐cfDNA and lower islet yield [−0.38 (0.18), *p* = .050] (Table 2).

Donor *miR*‐375 was highly predictive of recipient islet graft failure at 3 months [adjusted receiver operator curve (aROC) (SE): 0.813 (0.149)] with an optimal threshold value of <1.95 copies/nl predicting graft survival. Low levels of circulating donor *miR*‐375 also predicted a high level (≥90%) of islet viability [aROC (SE): 0.644 (0.166)] at an optimal threshold of <3.0 copies/nl.

In islet transplant recipients, low donor *INS*‐cfDNA levels predicted high islet yield [>500 000 IEQ; aROC (SE): 0.625 (0.155)] with an optimal threshold of <300 copies/ml but it was not predictive of 3‐month graft survival following islet transplantation [aROC (SE) 0.222 (0.147)].

Levels of the pro‐apoptotic miR‐34a were almost 10‐fold higher in IT‐donors, while there was no relationship between insulin use in donors and levels of other miRNA associated with beta‐cell death (Figure 1B). Levels of miR‐375 and miR‐34a were strongly correlated (Figure 1C).

Levels of IL‐6 were lower in IT versus no‐IT [0.7 (0.3‐1.3) vs. 3.0 (0.7‐7.2), *p* = .001] but levels of other apoptosis markers and inflammatory cytokines associated with beta‐cell death were not related to use of IT (Table S1).

## 4 DISCUSSION

We have shown that donor IT on ICU was associated with markers of beta‐cell death, lower beta‐cell function and higher glucose levels in donors. We also showed a relationship between a marker of beta‐cell death (miR‐375) and higher donor glucose levels. Finally, in a subgroup of pancreas donors providing islet grafts, higher donor miR‐375 levels predicted lower levels of islet viability at isolation and lower levels of graft function at 3 months.

To the best of our knowledge, this is the first study to assess the relationship between donor insulin use and beta‐cell death, glucose levels and beta‐cell function in the donor, and the first to link these parameters to islet isolation parameters and graft function in islet transplant recipients.

One study of elective total pancreatectomy and islet auto‐transplantation in 31 patients with chronic pancreatitis showed that higher pre‐operative circulating miR‐375 levels were associated with lower numbers of isolated islets and lower levels of graft function post‐transplantation.^14^ However, the authors did not show a relationship between miR‐375 and islet viability, although both are measures of cell death. However, it is only by considering the viability in the context of total isolated islets can the impact of donor beta‐cell death be properly interpreted.

Roels and colleagues recently showed the potential prognostic value of *miR*‐375, *INS*‐cfDNA and *GAD*‐65 measured in 39 islet transplant recipients 1‐h post‐transplantation.^15^ The authors reported that higher levels of recipient *miR*‐375 predicted adverse graft outcomes. They also reported superior predictive capabilities of *GAD*‐65 (aROC 0.83) and *miR*‐375 (aROC 0.77) over *INS*‐cfDNA. Our findings also suggest superiority of *miR*‐375 in predicting post‐transplant graft function and islet viability, but *INS*‐cfDNA was better at predicting islet yield. A crucial distinction between our work, and that of Roels et al. is that we measured these biomarkers in donor samples.

Finally, our study provides biomolecular mechanistic explanations for our previous findings that donor insulin use predicts graft dysfunction and graft loss in islet and pancreas transplantation.^1^, ^4^ Consequently, we ‘triangulate’ evidence^16^ supporting the hypothesis that beta‐cell death during the organ donation is associated with subsequent graft dysfunction following pancreas and islet transplantation.

Exogenous insulin requirement during organ donation could be the result of any combination of the following: (a) endogenous insulin deficiency caused by underlying diabetes (known or unknown); (b) irreversible beta‐cell death; (c) reversible beta‐cell dysfunction caused by short‐term metabolic stressors such as circulating cytokines; and/or (d) insulin resistance caused by inflammation, exogenous glucocorticoids and endogenous hormones such as catecholamines, which are elevated in brain death. Our data are consistent with the hypotheses: (a) that hyperglycaemia during organ donation occurs as a consequence of beta‐cell death; (b) that beta‐cell death predicts adverse outcomes in islet transplantation; and (c) that our clinical study findings^1^, ^4^ relating the donor insulin use to adverse transplant outcomes are explained by beta‐cell death.

In this study we have challenged the assumption that hyperglycaemia is driven by insulin resistance^3^ by showing the relationship between donor insulin use and markers of beta‐cell death. These data suggest that insulin resistance may be accountable for temporary and reversible hyperglycaemia, but that insulin insufficiency caused by impaired beta‐cell function and beta‐cell death might be a predominant driver behind hyperglycaemia. In practice, it seems plausible that the spectrum of beta‐cell stress (insulin resistance) culminating in loss of beta‐cell function and beta‐cell death (insulin insufficiency) could explain why some transplants from donors who required IT failed while others did not.

We showed strong relationships between measures of beta‐cell death and C‐peptide levels in donor samples. While elevated C‐peptide levels could be a consequence of enhanced beta cell function, it seems more plausible that the elevated C‐peptide levels have occurred as a consequence of beta cell death when these data are taken in the context of the other data presented.

We showed that lower levels of the pleiotropic inflammatory cytokine IL‐6 were associated with IT, which would be in keeping with the notion that IL‐6 may limit beta‐cell death. Supporting this idea, IL‐6 has been shown to have anti‐apoptotic properties in murine pancreatic islets and beta‐cell lines.^17^, ^18^, ^19^ In these studies, IL‐6 countered the effects of proinflammatory cytokines and promoted autophagy, while direct inhibition of IL‐6 led to increased beta‐cell death.^18^ However, an alternative explanation is that IL‐6 can increase beta‐cell apoptosis while preventing alpha‐cell apoptosis, thus leading to an overall protective effect on islets.^20^ This is important in the light of our data showing higher levels of the pro‐apoptotic miR‐34a in IT donors and in direct correlation with levels of miR‐375.

Here we make important progress towards developing objective markers of donor pancreas quality. *MiR*‐375, with its stable structure and half‐life of 8‐16 h,^21^ overcomes the limitations experienced by existing biomarkers. Meanwhile *INS*‐cfDNA provides a highly specific beta‐cell marker, which is independent of other measures of cellular function such as C‐peptide, which is released into the circulation upon cell death.^6^ We did not investigate alpha‐cell (glucagon secreting) death biomarkers such as GCG‐cfDNA, although this could be considered in future research. A combination of biomarkers representing each islet cell type and their function could permit a differential assessment of respective cell states.

Clinicians should also be aware that use of IT in donors predicts early beta‐cell dysfunction and islet failure 3 months after pancreas and islet transplantation. The use of biomarker data reflecting beta‐cell death in the context of clinical data reflecting poor glycaemic control in donors could be a powerful tool to aid clinical decision making.

There are no immediate clinical implications of our research. However, further research should assess whether combining information on donor insulin use and biomarkers of beta cell death can improve selection of appropriate pancreases for transplantation. It may be that donors using insulin with biomarker levels above specific thresholds (to be determined) are at such high risk of early failure that they are unsuitable for transplantation. More research is called for to assess whether such objective measures could help appropriately discard donor organs at high risk of failure and expand the donor pool by using organs previously considered unsuitable based on donor characteristics alone.

Our study has several strengths: First, we utilize data from the QUOD Biobank supporting novel mechanistic hypotheses that hyperglycaemia requiring IT in organ donors is driven by beta‐cell death. Secondly, we link QUOD data to islet isolation data and prospective functional data to show that donor *miR*‐375 and *INS*‐cfDNA predict islet viability and yield along with transplantation outcomes. Finally, we showed the potential clinical value of these data for improving the selection of pancreases for transplantation.

There are some limitations to our study. First, a large‐scale prospective study would be required to validate our findings, and to determine the feasibility and utility of using these measures of beta‐cell death to guide decision making in real time. Second, the time required to perform these assays may limit their immediate application into the clinical setting. It is conceivable that optimized assays based on current technology could influence clinical care in the setting of islet transplantation (~48 h from organ retrieval) but not presently pancreas transplantation (<10 h from organ retrieval). Third, we acknowledge the wide range of miR levels within organ donors and sought to address this in two ways by (a) performing both a digital PCR and a real‐time PCR assay, and (b) calculating the optimal thresholds by which clinically significant differences could be predicted. Organ donors are a heterogeneous population and, although we have accounted for factors that are known to be relevant, we cannot exclude the possibility of unmeasured confounding. Fourth, we recognize that the INS cfDNA assay did not provide a signal in 36 of the donor samples. The most probable explanation for this is that the sensitivity of the assay is related to the volume of the sample and we were restricted to 0.5 ml per assay. A greater volume of plasma sample would be required in future validation studies using the same assay. Finally, although the collection of donor samples was handled according to a regularly audited standardized operating procedure, we are not able to exclude the impact of variation in the handling of donor samples. Such factors include the times between sample acquisition, centrifugation, storage on ice and storage at −70°C, with the latter being determined by the distance from the donor hospital to the central processing laboratory. Newer technologies, such as the portable nanopore sequencing devices could overcome some of these issues and provide immediate data at the time of donation, which could ultimately guide decision making on use of donor organs.^22^

We limited the reporting of our post‐transplantation outcomes to the islet transplant cohort because (a) comprehensive assessment of beta‐cell function using a mixed meal tolerance test is not performed routinely following pancreas transplantation, and (b) technical factors such as thrombosis, pancreatitis and bleeding are accountable for a significant proportion of early graft losses following pancreas transplantation. This study was not powered to detect a relationship between markers of donor beta‐cell death and isolated islet failure following pancreas transplantation.

## 5 CONCLUSIONS

We provide data supporting the hypothesis that, in organ donors, hyperglycaemia requiring IT is a marker of beta‐cell death, poorer beta‐cell function, and is a predictor of islet isolation and transplant outcomes. Moreover, circulating cell‐free *miR*‐375 and *INS*‐cfDNA could have clinical utility in the objective assessment of donor pancreas quality in the setting of transplantation. Further prospective research is required to determine the utility of clinical data on IT and biomarker data on beta‐cell death and function to assess donor pancreas quality to guide donor selection in real time. Together, these measures could contribute towards expanding the organ donor pool, improving graft function and patient outcomes.

## AUTHOR CONTRIBUTIONS

IMS performed all aspects of the study and is lead author. AS performed all aspects of the study. JOS performed the study design, analysis and data interpretation relating to microRNA, and wrote the paper. CF analysed the data, performed statistical oversight and wrote the paper. NAH designed the study, performed oversight and wrote the paper. JC is Chair of the Pancreas Advisory Group at NHS Blood and Transplant with oversight over national data collection and wrote the paper. SF is Principal Investigator for islet transplantation (Edinburgh) and performed associated data collection and wrote the paper. MR is Principal Investigator for islet transplantation (Royal Free, London) and performed associated data collection and wrote the paper. PRVJ is Principal Investigator for islet transplantation (Oxford) and performed associated data collection and wrote the paper. PC is Principal Investigator for islet transplantation (Kings College, London) and performed associated data collection and wrote the paper. JB is Principal Investigator for islet transplantation (Bristol) and performed associated data collection and wrote the paper. JAMS is Principal Investigator for islet transplantation (Newcastle) and performed associated data collection; Chair of UK islet transplant consortium; wrote the paper. DN analysed and interpreted the data relating to cell‐free DNA, and wrote the paper. RS analysed and interpreted the data relating to cell‐free DNA, and wrote the paper. BG analysed and interpreted the data relating to cell‐free DNA, and wrote the paper. YD analysed and interpreted the data relating to cell‐free DNA, and wrote the paper. TA is deputy chair of pancreas advisory group at NHS Blood and Transplant with oversight over national data collection; study concept and oversight; and wrote the paper. MKR performed all aspects of the study, is Principal Investigator for islet transplantation (Manchester) and performed associated data collection; he is the joint senior author.

## FUNDING INFORMATION

Medical Research Council, Royal College of Surgeons of Edinburgh, Diabetes UK This research was supported by the NIHR Manchester Biomedical Research Centre (NIHR203308). The views expressed are those of the author(s) and not necessarily those of the NIHR or the Department of Health and Social Care.

## CONFLICT OF INTEREST STATEMENT

The authors have no conflicts of interest to declare.

### PEER REVIEW

The peer review history for this article is available at https://www.webofscience.com/api/gateway/wos/peer-review/10.1111/dom.15248.

## Supporting information


**DATA S1:** Supporting information.

## Data Availability

Raw data for miR and cfDNA is provided in the supplementary tables. All other data is reported in the article.
