# Supplementary material for: Beta‐cell death and dysfunction drives hyperglycaemia in organ donors
Source: Diabetes Obes Metab. 2023 Aug 30;25(12):3529–37. doi: 10.1111/dom.15248 (PMC10947469; doi:10.1111/dom.15248)
Supplement: Supplementary file 1 — DATA S1: Supporting information. [file DOM-25-3529-s001.docx]

Supplementary data

**Supplementary Table 1:** Levels of inflammatory cytokines and apoptosis markers comparing organ donors on intensive care treated with and without insulin therapy

| **Inflammatory cytokines and apoptosis markers** | **Insulin**  ng.hour/mL | **No insulin**  ng.hour/mL | **p-value** |
| --- | --- | --- | --- |
| Fas ligand | 39 (18-143) | 40 (14-131) | 0.608 |
| Granzyme B | 2.5 (1.4-4.1) | 2.9 (0.7-7.2) | 0.702 |
| IL1 | 1.4 (0.9-1.6) | 1.2 (0.8-0.7) | 0.581 |
| IL6 | 0.7 (0.3-1.3) | 3.0 (0.7-7.2) | 0.001 |
| TNFα | 61 (30-159) | 84 (36-187) | 0.277 |
| TRAIL | 0.07 (0.04-0.3) | 0.1 (0.04-0.3) | 0.558 |

Data are median (IQR)

IL – Interleukin; TNF – Tumour necrosis factor; TRAIL – TNF related apoptosis inducing ligand

**Supplementary table 2:** Raw data from levels of miR-375, *INS-*cfDNA and total cf-DNA

| **Subject ID** | **Insulin therapy** | **miR-375 copies/nL** | **INS-cfDNA copies/mL** | **Total-cfDNA ng/mL** |
| --- | --- | --- | --- | --- |
| 1 | no | 0.33 | 8.18 | 17.7 |
| 2 | no | 8.5 | 321.85 | 286 |
| 3 | no | 1.7 | 20.44 | 50.9 |
| 4 | no | 0 | 31.8 | 58 |
| 5 | no | 0.26 | 144.49 | 128 |
| 6 | no | 0.72 | 68.11 | 108 |
| 7 | no | 0.92 | 0 | 118 |
| 8 | no | 2.5 | 31.16 | 81.2 |
| 9 | no | 0.88 | 57.53 | 126 |
| 10 | no | 0.71 | 489.59 | 48.3 |
| 11 | no | 0.17 | 42.35 | 77.3 |
| 12 | no | 0 | 29.8 | 126 |
| 13 | no | 3.5 | 75.68 | 163 |
| 14 | no | 7 | 48.49 | 217 |
| 15 | no | 1.2 | 46.84 | 53.6 |
| 16 | no | 1.1 | 200.61 | 266 |
| 17 | no | 1.12 | 51.7 | 236 |
| 18 | no | 1.2 | 31.7 | 84.9 |
| 19 | no | 1 | 7.76 | 79.7 |
| 20 | no | 0.39 | 10.73 | 79.8 |
| 21 | no | 3.7 | 14.64 | 42.4 |
| 22 | no | 0.72 | 9.92 | 86.5 |
| 23 | no | 2.9 | 49.78 | 233 |
| 24 | no | 0.73 | 11.96 | 150 |
| 25 | no | 0.8 | 38.24 | 174 |
| 26 | no | 0.68 | 26.95 | 105 |
| 27 | no | 0.41 | 6.9 | 52.8 |
| 28 | no | * | 2.27 | 53.8 |
| 29 | no | 1.9 | 37.59 | 113 |
| 30 | no | 2 | 31.11 | 110 |
| 31 | no | 0.08 | 0.22 | 109 |
| 32 | no | 0.55 | 55.18 | 140 |
| 33 | no | 2.9 | 5.05 | 46.9 |
| 34 | no | 0 | 14.77 | 55.1 |
| 35 | no | * | 73.83 | 630 |
| 36 | no | 9.1 | 140.27 | 296 |
| 37 | no | 1.1 | 404.8 | 302 |
| 38 | no | 0.09 | 10.33 | 170 |
| 39 | no | * | * | * |
| 40 | no | 1.14 | 0 | 221 |
| 41 | no | * | 93.14 | 148.8 |
| 42 | no | 0.77 | 1.47 | 120 |
| 43 | no | 0 | 6.1 | 25.8 |
| 44 | no | 0.28 | 0 | 25.7 |
| 45 | no | 0.31 | 24.09 | 15.4 |
| 46 | no | 3.6 | * | * |
| 47 | no | 1.06 | * | * |
| 48 | no | 0.27 | * | * |
| 49 | no | 0.09 | * | * |
| 50 | no | * | * | * |
| 51 | no | * | * | * |
| 52 | no | * | * | * |
| 53 | yes | 2.6 | 51.3 | 89.9 |
| 54 | yes | 2.7 | 214.65 | 71.9 |
| 55 | yes | 3.3 | 102.65 | 179 |
| 56 | yes | 0.4 | 11.07 | 14.4 |
| 57 | yes | 0.17 | 72.48 | 66.1 |
| 58 | yes | 1.3 | 22.32 | 73 |
| 59 | yes | 1.4 | 45.06 | 101 |
| 60 | yes | 2.3 | 219.75 | 104 |
| 61 | yes | 1.5 | 13.18 | 109 |
| 62 | yes | 0.42 | 51.59 | 41.2 |
| 63 | yes | 0.07 | 2.76 | 88.1 |
| 64 | yes | 1 | 24.57 | 80.4 |
| 65 | yes | 0.1 | 280 | 95 |
| 66 | yes | 0 | 0 | 44.5 |
| 67 | yes | 0.75 | 99.18 | 213 |
| 68 | yes | 1.22 | 29.05 | 111 |
| 69 | yes | 1.4 | 13.41 | 53.6 |
| 70 | yes | 1.1 | 50.71 | 103 |
| 71 | yes | 0.28 | 46.77 | 171 |
| 72 | yes | 1.3 | 44 | 52 |
| 73 | yes | 0.81 | 22.7 | 57.6 |
| 74 | yes | 0 | 18.34 | 61.6 |
| 75 | yes | 0.18 | 23.99 | 80.4 |
| 76 | yes | 0.15 | 25.77 | 45.1 |
| 77 | yes | 1.5 | 15.39 | 50.3 |
| 78 | yes | 0.29 | 0 | 14.1 |
| 79 | yes | 2.5 | 33.43 | 98.8 |
| 80 | yes | 0.21 | 43.89 | 20.8 |
| 81 | yes | 3.5 | 48.97 | 351 |
| 82 | yes | * | 36.12 | 654 |
| 83 | yes | 3.1 | * | * |
| 84 | yes | 2.1 | * | * |
| 85 | yes | 0.18 | * | * |
| 86 | yes | 0.1 | * | * |
| 87 | yes | 0.08 | * | * |
| 88 | yes | * | * | * |
| 89 | yes | * | * | * |
| 90 | yes | * | * | * |
| 91 | yes | * | * | * |
| 92 | yes | * | * | * |

*denotes samples which did not return a measurable value
